# Alternative Biosorbents Based on Grape Pomace: Reducing Heavy Metals and Pesticides

**DOI:** 10.3390/toxics13050408

**Published:** 2025-05-17

**Authors:** Georgiana-Diana Gabur, Anamaria-Ioana Dumitrașcu, Carmen Teodosiu, Valeriu V. Cotea, Iulian Gabur

**Affiliations:** 1 Faculty of Horticulture, “Ion Ionescu de la Brad” Iasi University of Life Sciences, Aleea Mihail Sadoveanu nr. 3, 700490 Iasi, Romania; diana.gabur@iuls.ro (G.-D.G.); anaioanadum@gmail.com (A.-I.D.); valeriu.cotea@iuls.ro (V.V.C.); 2 Department of Environmental Engineering and Management, “Gheorghe Asachi” Technical University of Iasi, 73 Prof. Dimitrie Mangeron Street, 700050 Iasi, Romania; teodosiu.carmen@gmail.com; 3 Faculty of Agriculture, “Ion Ionescu de la Brad” Iasi University of Life Sciences, Aleea Mihail Sadoveanu nr. 3, 700490 Iasi, Romania

**Keywords:** grape pomace, biosorbent, heavy metals, pesticides, pollutants removal

## Abstract

Heavy metal and pesticide contaminations represent significant environmental and health hazards to humans and animals. Toxic heavy metals such as lead (Pb), cadmium (Cd), mercury (Hg), and copper (Cu) persist in the environment, bioaccumulating in beverages and food products from both natural and anthropogenic sources. Traditional remediation techniques, such as chemical precipitation and ion exchange, are effective but often costly and challenging to apply at a large scale. In recent years, grape pomace—a winemaking by-product rich in bioactive compounds—has emerged as a promising, low-cost biosorbent for the removal of such pollutants. Its high adsorption capacity, environmental friendliness, and availability make it a strong candidate for water and food decontamination processes. This study evaluates grape pomace and its biochar as sustainable biosorbents for heavy metal removal from water and soil, examining their adsorption efficiency, adsorption mechanisms, environmental benefits, advantages, limitations, and perspectives for future industrial-scale applications.

## 1. Introduction

Environmental pollution is a growing global issue that significantly affects human health, ecosystems, and biodiversity. Urbanization, population growth, consumerism, and industrial activities have contributed significantly to environmental pollution through the release of various contaminants, including heavy metal ions, polyfluoroalkyl substances (PFAs), volatile organic compounds (VOCs), persistent organic pollutants (POPs), and pesticides. Moreover, heavy metals and pesticide pollution are major environmental and health concerns, with significant consequences for both humans and ecosystems. Heavy metals such as lead (Pb), cadmium (Cd), mercury (Hg), and copper (Cu) are toxic elements that, once released into the environment, do not disappear and can persist for long periods of time, bioaccumulating in the food chain [1,2]. Heavy metals can come from two sources: natural and anthropogenic. They are present in soil due to pedogenetic processes, various chemical reactions occurring within the Earth’s crust, or from organic biological residues, typically existing at trace levels that are less toxic [3]. The principal anthropogenic sources of heavy metal contamination include industrial operations, mineral extraction, coal combustion in energy production facilities, utilization of fossil fuels, incineration of waste materials, inadequate management of electronic refuse, nuclear energy generation, application of pesticides and fertilizers in agricultural practices, vehicular emissions, and atmospheric deposition, among others [4,5]. These elements are inorganic contaminants with a high atomic weight (ranging from 63.5 to 200.6 Dalton) and a density greater than 5 g/cm^3^.

Long-term exposure to heavy metals has been associated with neurological, renal, and hepatic diseases, highlighting the importance of removing these contaminants from water, soil, and food [6]. Similarly, the overuse of pesticides in farming causes intense contamination of plant tissue, soil, environmental conditions, and even extends to water bodies [7]. Furthermore, the direct or indirect influences of pesticides on non-target organisms contribute to a disruption of the surrounding ecological balance because they are regarded as one of the most detrimental hazards that the ecosystem encounters; they can endure in the environment for extended periods, exhibiting carcinogenic properties [8]. Pesticides are persistent chemicals, having harmful effects on biodiversity and being associated with risks to human health, including endocrine disruption [9] and carcinogenic and mutagenic effects [10]. The unregulated application of pesticides results in their accumulation within trophic levels, thereby posing significant risks to non-target species, especially mammals [11]. The unregulated application of pesticides results in their accumulation within trophic levels, thereby posing significant risks to non-target species, especially mammals [11].

In light of these challenges, developing efficient and eco-friendly technologies for contaminant remediation is crucial. Conventional methods, such as absorption, flotation, chemical precipitation, ion exchange, or activated carbon treatment, are effective but can be expensive and difficult to apply on a large scale [12]. To overcome the limitations of these methods, researchers are increasingly focusing on biosorbent materials, which offer sustainable and cost-effective solutions for removing heavy metals and pesticides.

One promising material is grape pomace, which is an agro-industrial residue resulting from the winemaking process. Grape pomace is mainly composed of grape skins, seeds, and stems and contains several bioactive compounds, such as polyphenols, tannins, cellulose, and lignin, which exhibit a high adsorption capacity for heavy metals and pesticides [13].

According to the International Organization of Vine and Wine (OIV) data for 2024 [14], global wine production reached approximately 231 million hectoliters (mhL), with Europe accounting for the largest share at about 60% (139 mhL). In 2024, Italy is estimated to be the largest wine producer both in the European Union and globally, with a projected production of 41.0 mhL, marking an increase of 2.7 mhL (+7%) compared to 2023. France is expected to produce 36.9 mhL, while Spain remains the third-largest producer in the EU, with an estimated production of 33.6 mhL. Romania is the seventh-largest wine producer, with an estimated production of around 3.7 million hectoliters (mhL) in 2024.

Grape pomace constitutes about 30% of the total weight of the grapes used in winemaking [15], and as wine production increases, so does the volume of grape pomace. Improper disposal or incineration of this by-product may contribute to global warming or generate other challenges to the environment [16]. Given international environmental regulations [17], recycling and reusing grape pomace has become essential, presenting opportunities to produce high-value products such as energy, compost, animal feed, and other valuable materials.

The circular economy model is pivotal in managing grape pomace sustainably, reducing environmental pollution, promoting growth, and creating innovative product value systems. Grape pomace accounts for approximately 20–30% of the original grape weight, with global wine production generating an estimated 10 million tons of this organic waste annually [18]. This waste poses environmental challenges due to its low biodegradability, potential for acidification, and excess polyphenols that can harm soil and water quality.

Traditionally, grape pomace has been used for producing spirits, and prior to 2010, the EU required that it be delivered to distilleries for ethanol production. However, European Commission Regulation 5396/2008 expanded its uses to include organic fertilizers, animal feed, biomass for biogas production, and even pharmaceuticals and cosmetics, thanks to its potential health benefits [19].

Due to its abundance and biosorbent properties, grape pomace is being explored as an ecological alternative to conventional contaminant treatment methods. The high organic content of grape pomace makes it a suitable precursor for preparing activated carbon with specific surface characteristics [20]. Additionally, its use in recycling waste from the wine industry helps reduce pollution, disposal issues, and economic losses [21].

Another promising application of grape pomace is its conversion into biochar. Biochar is produced by pyrolyzing carbon-rich biomass sources under limited oxygen conditions [22,23]. Biochar has been studied extensively due to the wide range of properties it gains from different raw materials and modification treatments. Its adsorption performance has been analyzed, leading to significant advancements in using biochar for heavy metal and pesticide remediation [24]. These developments offer great potential for optimizing waste stream value and reducing environmental pollution [25].

Additionally, grape pomace can be converted into hydrochar, a product of hydrothermal carbonization, where biomass such as grape pomace is treated with hot water under high pressure. Hydrochar can be used for energy production, as a fertilizer, or as a contaminant removal agent [26,27].

This review aims to analyze the scientific literature on the use of grape pomace as a biosorbent material for reducing heavy metals and pesticides. We will explore the mechanisms by which grape pomace adsorbs these contaminants, its advantages and limitations, and possible future research directions, particularly regarding process optimization and industrial-scale applicability.

## 2. Methodology

VOS viewer is an innovative tool used to map bibliographic data and visually represent relationships based on keyword similarities. The bibliometric analysis results were visualized using VOS viewer software (version 1.6.20, Leiden University’s Centre for Science and Technology Studies (CWTS), The Netherlands), which generated a keyword co-occurrence network.

**Figure 1:**
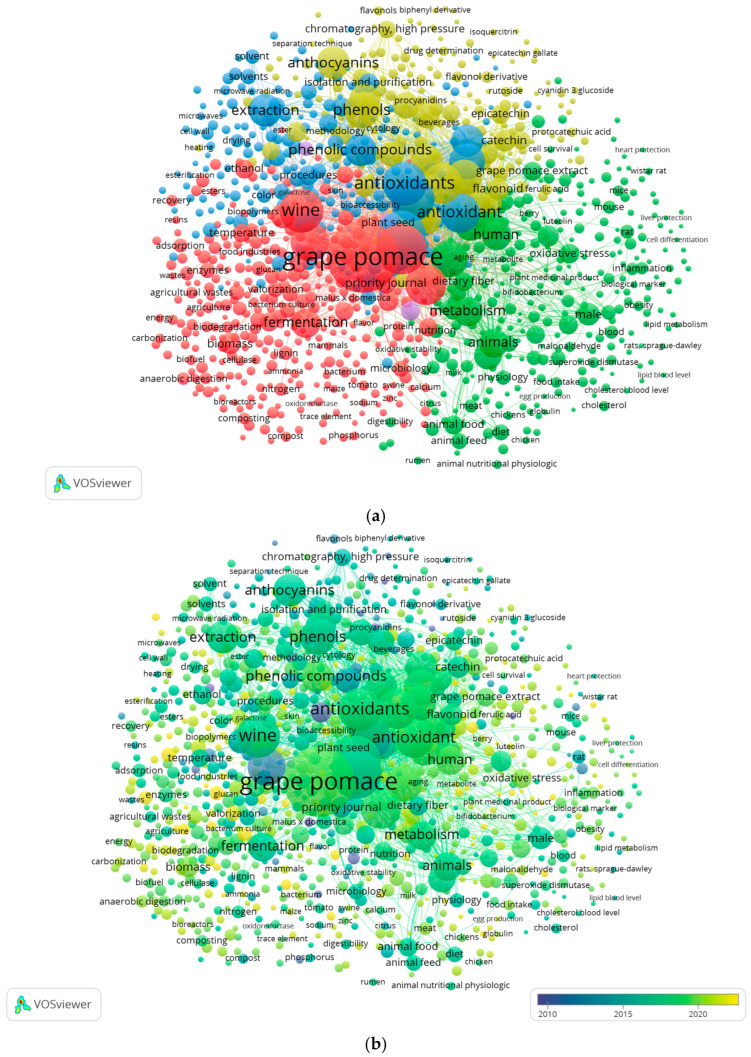
Network map representing grape pomace research from 2000 to 2025 created using VOS viewer: (**a**) cluster distribution of grape pomace research topics and trends; (**b**) temporal distribution of grape pomace research publications from 2010 to 2022.

The Scopus database indexed 2517 publications (including articles, reviews, conference papers, books, and book chapters) that included the phrase “grape pomace” in the keywords. The analysis focused on recent research topics related to grape pomace and identified five distinct research clusters, as shown in Figure 1a: Cluster 1 (red) contained 319 items, followed by Cluster 2 (green) with 285 items, Cluster 3 (blue) with 208 items, Cluster 4 (yellow) with 178 items, and Cluster 5 (purple) with 9 items. The sizes were based on their weight. The most frequent keyword was “grape pomace,” appearing 1152 times with a total link strength of 17,316, followed by “vitis,” with 538 occurrences and a link strength of 14,132, and “antioxidant,” with 435 occurrences and a link strength of 8646.

In addition, a trend analysis over time (Figure 1b) was performed to identify emerging research areas. The results showed that the first and second clusters (focused on topics such as valorization of grape pomace, biopolymers, phytochemicals, fruit waste, waste management, food ingredients, and bioaccessibility) include more recent studies (highlighted in yellow from 2022 onward).

## 3. Grape Pomace as a Biosorbent for Heavy Metals Removal

### 3.1. Biosorption Mechanisms for Pollutant Removal from Water

Biosorption is an important aspect of sustainable development and is classified under biotechnological approaches [28]. It is considered an eco-friendly, cost-effective, and efficient method for water treatment [29]. This technique helps reduce the concentration of water pollutants to levels within the acceptable limits set by federal regulations [30]. Moreover, it aligns with the principles of green chemistry.

In essence, biosorption is a metabolism-independent process (passive uptake) that uses biowastes to remove pollutants from water. Recycling these biomasses offers several benefits, including waste reduction. Their use, whether in their natural or modified forms, directly contributes to minimizing waste, addressing numerous ecological and environmental challenges [31]. Moreover, this process presents notable advantages, including low operational and production costs, along with high efficiency. Sorption is generally defined as a physicochemical process in which the molecules of a substance (sorbate) adhere to the surface of another material (sorbent). The efficiency of sorption is influenced by factors such as solubility, molecular size, surface charge, chemical composition, reactivity, and hydrophobicity. In particular, the presence of various functional groups on the sorbent enhances the uptake of both metal ions and dyes. Among the primary mechanisms involved are electrostatic interactions, ion exchange, physical adsorption, and the formation of complexes between metal cations and ligands located within the biopolymer’s cell wall structure.

The interaction between water pollutants and biosorbents primarily occurs in two different forms: surface sorption and interstitial sorption. In the first case, in surface sorption, sorbate molecules move from the aqueous solution to the biosorbent surface. Once pollutants (molecules and/or ions) pass through the boundary layer surrounding the biosorbent, they bind to the active sites on its surface, effectively removing them from the solution. This type of sorption typically occurs through dipole interactions, hydrogen bonding, or Van der Waals forces. In contrast, the second case, interstitial sorption, involves the diffusion of pollutants (molecules and/or ions) into the pores of the biosorbent (macro-, meso-, or micropores). Once inside, the pollutants are biosorbed onto the interior surface of the biosorbent [32]. Furthermore, electrostatic interactions play a key role in removing various pollutants from water and wastewater, serving as a major contributor to biosorption. The high density of functional groups (e.g., hydroxyl, carboxyl, amino, and phosphate) on the biosorbent surface enhances pollutant capture and is strongly pH-dependent.

Beyond physical sorption, biosorption also involves various chemical interactions. Complexation is a key mechanism wherein metal ions bind with ligands—molecules that possess lone electron pairs—to form metal–ligand complexes. These can be mononuclear (single metal ion) or polynuclear (multiple metal ions), with the overall charge depending on the nature of the ligands. Chelation, a specific form of complexation, involves multidentate ligands forming stable ring structures with metal ions, often leading to enhanced adsorption stability [33].

Ion exchange is another prominent biosorption mechanism that is widely used in water treatment. It involves the substitution of naturally occurring ions on the biosorbent surface with target metal ions from the aqueous phase. This can occur via cation or anion exchange, depending on the ionic species involved. For instance, carboxyl groups may act as cation exchange sites, enabling the removal of heavy metals such as Pb^2+^ or Cd^2+^ through displacement reactions [34].

Adsorptive deposition is also widely recognized, particularly in the removal of heavy metals. In this process, metal ions exhibit a high affinity for functional groups present on biosorbent surfaces—often derived from microbial biomass or plant-based residues [35]. Upon adsorption, metal ions may undergo reduction reactions, leading to the formation of zero-valent metals via nucleation and crystallization. This phenomenon is especially relevant for metals such as mercury and platinum.

An important application of this redox-based mechanism is seen in the detoxification of hexavalent chromium (Cr (VI)), which is a highly toxic and environmentally persistent contaminant. Biosorption not only facilitates the adsorption of Cr (VI) but also promotes its reduction to trivalent chromium (Cr (III)) or elemental chromium, both of which are significantly less toxic. This transformation plays a crucial role in reducing the environmental risks associated with chromium-laden effluents [36].

**Figure 2:**
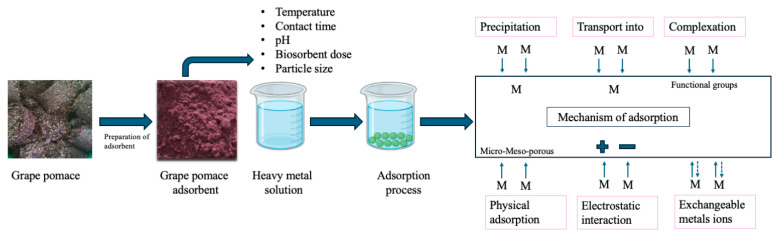
Schematic representation of the adsorption mechanisms involved in heavy metal removal by grape pomace-derived biosorbents.

A schematic overview of the key biosorption mechanisms is presented in Figure 2.

The technique is highly cost-effective, as it involves a straightforward process that requires no additional nutrients and consumes no energy. Bioadsorption refers to the removal of heavy metals from liquids using biological materials such as bacteria, algae, or plant-based waste like grape pomace [12]. Moreover, it is an affordable option that can be completed in a very short time. Additionally, biosorbents are recyclable, and various types can be specifically tailored to target particular metals of interest.

The ‘Waste to Resource’ concept emphasizes the conversion of waste materials into valuable products through methods such as recycling, upcycling, and repurposing. Rather than directing waste to landfills or incineration, this approach aims to recover and reintegrate useful materials into the production cycle. This strategy not only reduces the volume of waste but also conserves natural resources and mitigates the environmental impacts associated with conventional disposal methods. Additionally, waste-to-resource initiatives create new economic opportunities and offer a sustainable supply of raw materials for various industries. As a fundamental principle of the circular economy, this concept supports a closed-loop system in which resource efficiency is maximized and waste generation is minimized.

A comparative evaluation of grape pomace (GP) preparation methods reported in the literature reveals a core sequence of steps commonly employed: washing, drying, grinding, and particle size selection. These fundamental processes are often adapted by researchers to meet the requirements of specific applications, such as heavy metal adsorption or wine fining. Oliveira et al. [35] prepared GP biosorbents by washing the biomass with distilled water, drying it at 45 °C to constant weight, and selecting particles between 0.14 and 1.4 mm after mechanical grinding. This relatively mild thermal treatment preserves functional groups while ensuring particle uniformity. A more complex approach is described by Kumar et al. [36]. The pomace was dried at higher temperatures (80–100 °C for 48 h), ground, and subjected to successive washing, vacuum filtration, sedimentation, and regrinding to yield a fine powder capable of passing through a 100-mesh sieve. This multistep procedure likely increases purity and surface area, enhancing adsorption potential [36]. Another approach used freshly pressed GP with an initial moisture content of 76%, which was oven-dried at 100 ± 5 °C for 24 h, ground with a food-grade grinder, and stored for further application [20]. Their method aligns with scalable industrial processes while preserving material functionality. In contrast, Kokkinomagoulos and Kandylis [37] incorporated a unique ethanol-based double maceration extraction (70% *v*/*v* ethanol, 1:3 *w*/*v*, 150 rpm, 20 °C, 24 h) before drying to selectively remove phenolic compounds and expose binding sites. The treated pomace was then freeze-dried and finely milled to <0.24 mm. This protocol is particularly suitable for applications in wine fining, where the selective removal of polyphenols is desirable.

Despite variations in temperature, equipment, and post-processing, all reviewed methods share a similar preparatory framework. However, the choice of drying technique (low-temperature, high-temperature, or freeze-drying), pretreatment steps (e.g., ethanol extraction), and final particle size are critical variables that significantly influence the biosorptive performance and application suitability of GP-derived biosorbents [38].

Future studies should focus on linking specific preparation parameters to adsorption efficiency for different contaminants or processing goals. Comparative studies under standardized conditions are needed to establish optimal protocols tailored to industrial, environmental, or enological applications [39].

In addition, green synthesis is an emerging and sustainable approach that utilizes renewable resources to extract biologically active compounds and produce nanoparticles with tailored properties. By eliminating the use of hazardous chemicals and toxic solvents, this method minimizes environmental impact and waste generation. It is also cost-effective, scalable, and suitable for large-scale production. Additionally, the biocompatible materials obtained through green synthesis hold significant potential for biomedical applications. Overall, green synthesis has the potential to revolutionize nanoparticle production, contributing to a more sustainable future.

Table 1 presents the use of waste biomass as a biosorbent for heavy metal removal. Similarly, biosorbents are considered cost-effective due to their low cost. Numerous studies have evaluated the effectiveness of these materials as biosorbents [40].

An economical and sustainable solution to heavy metal pollution in wastewater is the use of by-products such as spent brewer’s yeast and rice hulls as biosorbents. This approach is particularly beneficial for industries, including the food sector, that generate significant amounts of waste and by-products. Studies have shown that rice hulls can effectively remove heavy metals such as Ni, Cd, Cu, Pb, and Zn from metal-contaminated solutions under optimal conditions. Biosorbents derived from agricultural waste are also promising for wastewater treatment, particularly for the removal of heavy metals. The cost-effectiveness and potential of these materials have been extensively studied. Examples of agricultural-waste-derived biosorbents include *Portulaca oleracea*, orange peel, *Annona squamosa* peel, tea waste, neem biomass, and potato peel waste. These materials exhibit a high affinity for heavy metals binding attributed to the presence of diverse functional groups that facilitate metal sequestration [41]. In addition to their effectiveness, agricultural waste biosorbents offer an environmentally friendly solution with no associated health risks, making them a sustainable alternative for wastewater treatment applications.

Grape pomace, as a biological waste, has shown significant promise as a biosorbent for removing toxic substances, including pesticides and heavy metals, thanks to its high content of functional groups capable of interacting with pollutants.

Copper (Cu), lead (Pb), nickel (Ni), chromium (Cr), arsenic (As), iron (Fe), and cadmium (Cd) have been successfully removed from aqueous solutions using agricultural residues derived from various waste materials. The adsorption process follows a pseudo-second-order kinetic model and fits the Langmuir isotherm model. Table 1 provides a summary of the use of biomass-based agricultural residues for heavy metal removal.

**Table 1:** Utilization of waste biomass as an adsorbent for the removal of heavy metals.

Biosorbent	Heavy Metals	Parameters				Kinetics/ Isotherm	Adsorption Capacity	References
		Dose	pH	Time	Temp (°C)			
Green tea waste	As (III)	0.3 g	3	3 h	33	Pseudo-second-order/ Langmuir	0.4212 mg/g	[32]
	Ni (II)		7				0.3116 mg/g	
Plum waste	Cr (III)	4 g/L	6	30 min	22	Pseudo-second-order/ Langmuir	14.024 mg/g	[42]
	Pb (II)						12.689 mg/g	
Apricot waste	Cr (III)	4 g/L	6	30 min	22	Pseudo-second-order/ Freundlich	28.799 mg/g	[42]
	Pb (II)						23.890 mg/g	
Banana peels	Pb (II)	0.1 g	6	24 h	25	Pseudo-second-order/ Freundlich	241 mg/g	[43]
Pomegranate peel	Cd (II)	0.1 g	5	480 min	25	Pseudo-second-order/ Langmuir	132.5 mg/g	[44]
Litchi peel	Cd (II)	0.1 g	5	480 min	25	Pseudo-second-order/ Langmuir	230.5 mg/g	[44]
Lemon peel	Ni (II)	5 g/L	5	180 min	25	Pseudo-first-order/ Langmuir	36.74 mg/g	[45]
Jackfruit seed waste	Mn (VII)	1 g/L	7	-	35	Pseudo-second-order/ Langmuir	79.8 mg/g	[46]
	Pb (II						79.9 mg/g	
	Cu (II)						97.9 mg/g	
	Cd (II)						79.4 mg/g	
	Fe (III)						76.4 mg/g	
Dragon fruit peel	Pb (II)	0.25 g/L	4	3 h	60	Pseudo-second-order/ Langmuir	97.087 mg/g	[47]
	Cd (II)						86.207 mg/g	
Melon peel	Cu	0.0015 g/L	6	1 h	30	Pseudo-second-order/ Langmuir	77.76 mg/g	[48]
	Pb						191.93 mg/g	
	Cd						76.16 mg/g	
Watermelon rind	As (III)	1 g/L	8.2	72 h	65	Pseudo-first o-der/ Langmuir	99%	[49]
	As (V)		4.6				98%	
Cabbage leaves	Cd (II)	0.5 g	6	150 min	50	Pseudo-second-order/Langmuir	88.92%	[50]
	Cu (II)						92.42%	
	Pb (II)						95.67%	
Soy waste biomass	Ni (II)	5.0 g/L	5.5	4 h	20	Pseudo-second-order/ Freundlich	128%	[51]
	Cu (II)						131%	
	Pb (II)						196%	
Waste biomass biochar	Fe	1.0 g/L	6	20 min	450	Pseudo-second-order/Langmuir	10.95%	[52]
	Ni						99.01%	
	Cu						91.60%	
	Cr						99.06%	
	Cd						99.24%	
	Pb						95.52%	

### 3.2. Heavy Metal Removal from Aqueous Solutions

In this section, the influence of different parameters such as contact time, adsorbent dose, pH, and temperature on the ability of grape pomace-derived adsorbents to remove heavy metals from different liquid solutions is investigated.

#### 3.2.1. Lead and Cadmium Removal

Lead (Pb) is a hazardous heavy metal and constitutes an omnipresent contaminant in both terrestrial and aquatic ecosystems. The principal sources of Pb in aquatic environments are drainage and surface runoff effluents discharged by industrial activities. Lead can be effectively removed through adsorption using a variety of natural adsorbents.

Evaluation of four distinct biomaterials derived from oenological waste for their efficacy in lead (Pb) removal from wastewater included the following: unprocessed grape marc from Merlot variety (MR), Sauvignon Blanc variety (SbR), biorefined grape marc Merlot variety (ME), and Sauvignon Blanc variety (SbE) [53]. At a dosage of 0.50 g/L biosorbent, an initial Pb concentration of 20 mg/L, and optimal pH 5.5 level, the removal efficiencies were 71% (MR), 78% (SbR), 80% (ME), and 97% (SbE). Langmuir isotherm modeling revealed maximum adsorption capacities of 40.14 mg/g for ME and 63.76 mg/g for SbE, indicating that grape marc—a common agricultural waste—holds promise as an accessible and low-cost adsorbent for lead remediation in water [53].

Hydrochar was investigated by Petrović et al. [54] as a new adsorbent for Pb^2+^ adsorption from aqueous solution. In this study, hydrochar was synthesized from grape pomace through hydrothermal carbonization. Grape pomace served as the biomass precursor, and the resulting hydrochar was further modified with alkali to enhance its capacity for adsorbing Pb^2+^ ions from aqueous solutions. Activation with 2 M KOH significantly enhanced its adsorption capacity from 27.8 mg/g to 137 mg/g at pH 5. Mechanisms such as ion exchange, chemisorption, and Pb^2+^–π interactions were identified as key contributors. The Sips isotherm model best fit the experimental data, further confirming the material’s efficiency.

In a recent study, the authors conducted an adsorption experiment of Pb (II) in water using biochar produced from grape pomace and its second-stage by-products [55]. In this study, a 0.025 g biochar dosage was used for 25 mL aliquot of Pb solution with pH = 6.50. The lignin component of the grape pomace pyrolyzed at 700 °C adsorbed the most Pb (II), with a removal rate of 66.5% at the initial concentration of 300 mg/L over a duration of 30 min. In another study, related to Cd (II) removal, Kumar et al. [36] optimized adsorption using powdered grape pomace. The suitability of the equation was evaluated by the determination coefficient (R^2^ = 0.99916). Optimal conditions for maximum Cd (II) removal efficiency were identified as a pH of 5.2, a grape pomace dosage of 12.5 g/L, a temperature of 28.5 °C, and an initial metal concentration of 43 mg/L. Moreover, Melia et al. [56] explored the adsorption mechanisms for cadmium, finding interactions with oxygen, sulfur, and carbonate groups—likely involving polyphenol-derived functionalities—and possible calcium displacement. This study results demonstrates that grape waste is an effective unprocessed biosorbent for Cd removal.

Furthermore, Trakal et al. [57] noted that magnetizing wheat straw and grape biochar did not enhance Pb (II) adsorption but significantly improved Cd (II) removal, even in multimetal systems [58]. Surface complexation with hydroxyl and carboxyl groups was suggested as a key factor influencing adsorption behavior.

Complementary experimental results have also demonstrated that the form of fruit pomace significantly influences its capacity to remove Cd (II) and Pb (II) from aqueous solutions. Freeze-dried pomace was more effective, likely through increased porosity [59]. The porous microstructure of freeze-dried materials allows for high water absorption but exhibits low mechanical resistance [60,61]. In contrast, convection drying results in significant shrinkage, reduced porosity, and lower sorption efficiency [62]. Among the convection-dried pomace samples, grape pomace exhibited the highest adsorption efficiency. Notably, most freeze-dried pomace samples were over 50% more efficient at removing cadmium (II) ions compared to convection-dried pomace, except for grape marc, which maintained similar adsorption levels.

Furthermore, lead consistently demonstrated a significantly higher adsorption affinity than cadmium, consistent with the findings of Krol et al. [63], who reported over 98% lead removal efficiency. Farinella et al. [64] similarly observed superior adsorption by grape pomace relative to cadmium and lead. Optimal adsorption conditions differed for the two metals: maximum cadmium uptake occurred at pH 7.0, while lead was most effectively adsorbed at pH 3.0. In both cases, equilibrium was reached within 5 min of contact time. For this study, the removal efficiency of Cd (II) was approx. 95.8%, while for Pb (II), it was 40.8%. Although lead adsorption proceeded more slowly, it was ultimately more effective—likely due to the smaller Stokes radius of hydrated lead ions (0.297 nm) compared to cadmium (0.393 nm), which facilitates greater access to active sites on the biosorbent surface [65].

Grape pomace has great potential as a precursor for wastewater treatment, particularly for heavy metal removal [20]. Oven-dried grape pomace (GP) showed limitations, such as lower metal ion binding, while thermal treatment enhanced porosity and selective surface sites, improving the maximum adsorption capacity (GPMAC). The developed GPMAC effectively minimized secondary pollution and demonstrated superior adsorbent performance. Batch adsorption studies indicated that maximum adsorption occurred at a contact time of 120 min, pH of 5, adsorbate concentrations of 200 mg/L, and a temperature of 45 °C. According to the Langmuir model, the adsorption capacities were 357.14 mg/g for Pb (II) and 156.25 mg/g for Cd^2+^. FTIR and pH analyses identified electrostatic attraction as the primary binding mechanism. The adsorption process was influenced by both the surface chemistry and textural properties of the GPMAC, and thermodynamic studies confirmed its feasibility and spontaneity. The biosorbent demonstrated 94% efficiency after five cycles, indicating its potential for long-term use in real industrial wastewater treatment.

Interestingly, grape stalk waste is an effective sorbent for removing Pb (II) and Cd (II) ions from aqueous solutions at near-neutral pH [66]. Adsorption experiments using grape stalks as biosorbents demonstrated promising maximum sorption capacities for both lead and cadmium under slightly acidic conditions (pH ~5.5). The recorded adsorption capacities were 0.241 mmol g^−1^ for Pb (II) and 0.248 mmol g^−1^ for Cd (II), indicating a comparable and efficient uptake of both metal ions. At an initial metal concentration of 0.5 mmol per gram of adsorbent, these capacities correspond to estimated removal efficiencies of approximately 48.2% for lead and 49.6% for cadmium. Kinetic modeling indicated that the process followed a pseudo-second-order mechanism. The presence of NaCl and NaClO_4_ significantly reduced cadmium uptake, with a lesser effect on lead. Competing metal ions further inhibited Cd (II) sorption, while Pb (II) remained largely unaffected. Desorption tests showed complete removal of Pb (II) with HCl or EDTA, whereas Cd (II) desorption reached approximately 65%. These results indicate that, beyond ion exchange, surface complexation and electrostatic interactions contribute to the metal sorption process. Moreover, Miralles et al. [67] conducted fixed-bed column experiments to assess the potential of grape stalk waste for the removal of Pb (II) and Cd (II) ions from aqueous solutions. The results demonstrated that the sorption capacities and transport parameters were influenced by the initial metal concentration and sorbent particle size. The study utilized the Thomas model and the CXTFIT code to analyze the transport and sorption parameters, highlighting the practical application of grape stalks in wastewater treatment.

Biochars derived from grapevine shoots have a role in enhancing the removal of heavy metals [68]. Biochars pyrolyzed at temperatures of 300 °C, 450 °C, and 600 °C demonstrated outstanding adsorption capacities for cadmium (250.81, 327.31, and 377.36 mg/g, respectively) and lead (156.12, 237.25, and 258.86 mg/g, respectively). The study revealed that effective adsorption required a solution pH greater than 3, with ion exchange, surface complexation, and co-precipitation being the primary mechanisms for heavy metal removal. The data confirmed that pyrolyzed grapevine shoots could be a suitable material for producing biomass-based adsorbents for the removal of heavy metals from wastewater. Sardella et al. [69] found that grape pomace activated carbon has a 98% removal for lead and cadmium at pH 5.5 and 6, respectively, at 800 °C for 105 min. The initial lead concentration was 1.31 mg/L, while cadmium was under the detection limit. The results indicated lead removals around 50% for GP-AC and GS-AC, while for GL-AC, it was only 11%.

In summary, the studies presented above have demonstrated the significant potential of grape-derived biomaterials—such as grape marc, pomace, stalks, hydrochar, and biochar—as effective and sustainable adsorbents for removing heavy metals like lead (Pb) and cadmium (Cd) from aqueous solutions. These materials, which are typically agricultural by-products from the winemaking process, present a low-cost and eco-friendly approach to water remediation. Moreover, the adsorption efficiency of these grape-based materials is significantly enhanced through thermal and chemical treatments. Processes such as pyrolysis and activation (particularly with potassium hydroxide—KOH) modify the surface area, porosity, and functional groups of the materials, resulting in improved metal-binding capabilities. Among the treated materials, activated hydrochar has shown particularly high adsorption capacities, with reported values up to 137 mg/g for cadmium and 64 mg/g for lead. On the other hand, multiple studies highlight that lead tends to be more effectively adsorbed than cadmium. This is attributed to the smaller hydrated ion radius of lead, which allows for stronger interactions with the adsorbent surfaces. Additionally, adsorption is influenced by environmental conditions, with optimal removal occurring under slightly acidic pH levels, typically around 5.5. The contact time required for efficient adsorption varies between 5 and 120 min, depending on the specific type of adsorbent and the metal involved. The Langmuir adsorption isotherm model frequently provided a good fit for experimental data, supporting the high adsorption capacities of these materials. Additionally, surface mechanisms such as electrostatic attraction, ion exchange, and complexation with hydroxyl and carboxyl groups were identified as key drivers of the adsorption process. The sustainability of these materials is also a key advantage, as the adsorbents retained a significant portion of their effectiveness after multiple cycles, and many could be reused for lead recovery. These findings demonstrate the practicality of using grape pomace and related by-products in wastewater treatment for heavy metal remediation.

#### 3.2.2. Nickel Removal

Wine processing waste sludge (WPWS) has been employed as an effective adsorbent for the removal of Ni, which is attributed to its high organic matter content, rough surface morphology, and abundance of amino and carboxyl functional groups. The adsorption capacity is significantly influenced by factors such as pH, temperature, initial nickel concentration, and particle size of the waste material. According to the Langmuir isotherm model, the maximum sorption capacity was determined to be 66.55 mol/g at 50 °C. Further investigation should be conducted to explore the interactions between the functional groups of WPWS and nickel [70].

Winery by-products, such as grape stalks, have been studied as adsorbents for heavy metals like Ni, Cu, and Cr in aqueous solutions. The results indicate that ion exchange plays a key role in the sorption process, with a significant release of Ca^2+^, Mg^2+^, K^+^, and H^+^ ions from the grape stalks as they absorb Cu (II) and Ni (II). Furthermore, grape stalks can be reused for the treatment of metal-containing effluents according to Villaescusa et al. [71].

#### 3.2.3. Copper Removal

Grape bagasse chars have a composition of cellulose (3.94%), lignin (41.51%), hemicellulose (12.37%), and >70% volatile matter content, which renders them highly suitable for char production. The pyrolysis procedure, with varying temperatures, resulted in the formation of the gaseous fraction in the range of 20–33%, with H_2_ being the predominant gaseous product. Pyrolysis under any condition yielded approximately 31% char. The synthesized chars at the temperature levels of 700, 800, and 900 °C showed characteristic textural properties; however, they underwent the same surface chemistry with comparable adsorption efficiency in the adsorption of Cu (II). Accordingly, it was established that the char synthesized at 700 °C (C700) is most effective in the adsorption of Cu (II) at pH 5.5 with an adsorbent dose of 1.5 g L^−1^, with initial Cu (II) concentrations ranging from 30 to 200 mg L^−1^. These conditions yielded a percentage removal of >95%. The maximum adsorption capacity was quantified at 42 mg/g, which is a striking result compared to the literature [72].

#### 3.2.4. Chromium Removal

Grape waste has been investigated for its potential in the adsorption and separation of hexavalent chromium (Cr (VI)) ions from aqueous environments. The cross-linked gel derived from grape waste has demonstrated a selective affinity for Cr (VI) ions, proving to be effective in their removal from synthetic aqueous solutions. The adsorption of Cr (VI) was dependent on pH, with peak adsorption occurring at a pH level of 4. Furthermore, the adsorption capacity was observed to increase with increasing solute concentration, as articulated by the Langmuir adsorption model. The maximum adsorption capacity for Cr (VI) reached 1.91 mol/kg at pH 4. The mechanism of Cr (VI) removal was identified as an esterification reaction, which was corroborated by Fourier transform infrared (FTIR) spectroscopy measurements [73].

#### 3.2.5. Mercury Removal

Mercury is an incredibly harmful heavy metal for humans and ecosystems [74].

Grape bagasse chars were synthesized under varying pyrolysis conditions, which were optimized to enhance mercury adsorption from aqueous solutions. The mercury adsorption process was endothermic, achieving a maximum adsorption capacity of 45.9 mg/g. Mercury removal increased with increasing temperature and pH, with the best value observed at a pH of 4 and 40 °C [75].

In relation to the adsorption of Hg (II), it was determined that optimal adsorption takes place at a pH of 5 to 7. In this study, two biosorbents derived from green grape marc, generated during Negroamaro wine production, were effectively utilized for the adsorption of Hg (II) ions in neutral aqueous solutions. The environmentally friendly methodology used in the preparation of these biosorbents allowed for the positive endorsement of green chemicals such as water, ethanol, and citric acid. Notably, the adsorption isotherm analysis indicated that the GM-CA biosorbent, which was synthesized using citric acid, exhibited a maximum adsorption capacity of 36.39 mg/g that was facilitated by a physical adsorption mechanism of Hg (II) onto the matrix. Ultimately, it is pertinent to emphasize that these findings may pave the way for future research aimed at developing innovative green adsorbents for the removal of Hg (II) ions from potable water sources [76].

#### 3.2.6. Nickel and Zinc Removal

A real industrial effluent originating from pretreatment and painting processes was treated using biochar derived from Serra Gaúcha as an adsorbent. The biochar was produced in a pilot-scale plant from composted grape pomace. The biochar showed an equilibrium between acidic and basic groups on the surface. The presence of irregular cavities and mesopores in the biochar structure was confirmed through N_2_ physisorption analysis and scanning electron microscopy (SEM). In the industrial effluent, nickel (Ni) and zinc (Zn) were identified as the primary contaminants. Adsorption equilibrium for both metals was achieved within 60 min without the need for pH adjustment. The adsorption process was found to be endothermic, favorable, and spontaneous. These findings confirm the effective removal of Ni and Zn from the industrial effluent, with final concentrations meeting the regulatory limits for disposal on agricultural soil [77].

Zinc (II) removal from the solution was lowest at a pH of 2.0 (29%), likely due to competition between metal ions and H_3_O^+^ for adsorption sites. Increasing the pH from 3.0 to 6.0 resulted in a relatively stable removal efficiency (~43%), with the highest adsorption capacities observed at pH 3.0 and 5.0. This suggests a broad range of suitable pH values for zinc (II) removal. However, at pH levels above 6.0, metal precipitation occurs, which is undesirable for adsorption. The adsorption process was also influenced by temperature, with zinc (II) removal decreasing from 45% to 37% as the temperature increased from 22 °C to 52 °C. This indicates that lower temperatures favor adsorption under the tested conditions (100 mg/L initial concentration, 20 mg biosorbent mass, pH 3.0, and 150 rpm stirring) [78]. This decrease in surface activity with rising temperature suggests that the adsorption of zinc (II) onto grape bagasse is an exothermic process. This implies that lower temperatures favor adsorption, as higher temperatures may reduce the interaction between zinc (II) ions and the biosorbent, leading to decreased adsorption efficiency [79].

#### 3.2.7. Grape Pomace for More Heavy Metals Removal

In a Cd (II), Ni (II), Co (II), and Pb (II) aqueous solution, Pb (II) exhibited the highest biosorption percentage across all studied biosorbents. For this specific metal, the retention capacity was found to be superior for Palomino Fino grape seed (89%) and Cabernet Sauvignon grape pomace (66%) [80].

Pisco grape pomace extract had a lower metal chelating ability for Cu^2+^ than for Fe^2+^ [81].

Studies by McKay et al. [82] and Xu et al. [83] demonstrated that various biochar types are efficient for removing heavy metals such as cadmium (Cd), copper (Cu), and zinc (Zn) from aqueous solutions. Biochar types derived from nut shells, plum stones, wheat straws, as well as grape stalks and husks exhibited significant effectiveness in cadmium removal from aqueous solutions, with wheat straw and grape husks and stalks demonstrating the highest efficiency [84].

Table 2 summarizes the conditions and maximum absorption capacities for the absorption of heavy metals from aqueous solutions.

Grape pomace-derived materials have demonstrated significant potential as low-cost and effective adsorbents for the removal of various heavy metals from aqueous solutions. The effectiveness of these materials is influenced by several factors, including contact time, adsorbent dose, pH, and temperature. Studies have shown that different forms of grape pomace, such as raw grape pomace, biochar, and hydrochar, exhibit varying degrees of success in removing heavy metals like lead (Pb), cadmium (Cd), nickel (Ni), copper (Cu), chromium (Cr), and mercury (Hg).

For instance, lead removal has shown promising results, with maximum adsorption capacities reaching up to 64 mg/g for biorefined grape pomace. Similarly, cadmium and copper removal have been effectively achieved through the use of biochars and grape pomace-based materials, demonstrating high adsorption capacities and efficiency. Nickel and zinc were also successfully removed from industrial effluents using biochar derived from grape pomace, with favorable outcomes at moderate pH levels.

Moreover, the mechanisms behind the adsorption of these heavy metals include electrostatic attraction, ion exchange, chemisorption, and surface complexation, which are often influenced by the chemical properties and surface morphology of the adsorbents. Thermal and chemical modifications, such as pyrolysis and activation, were found to enhance the adsorption capacities of grape pomace-based adsorbents.

The studies collectively highlight the versatility and effectiveness of grape pomace in addressing heavy metal contamination in water and beverages, supporting its potential as an environmentally friendly and cost-effective solution for water purification, wastewater treatment, and wine production. While studies on the use of grape pomace to remove heavy metals and pesticides from beverages are limited, there is sufficient evidence to suggest its potential as an effective adsorbent. Further research is needed to fully understand its capabilities and optimize its application in beverage purification.

### 3.3. Grape Pomace and Biochar for Reducing Heavy Metal Bioavailability in Soils

The literature on the use of grape pomace for the removal of heavy metals from soil is limited.

Research conducted by Karimi et al. [86] explored the effects of biochar on various physiological parameters of grape plants, including relative water content, ion leakage, chlorophyll, phosphorus, and rhizosphere soil cadmium availability under cadmium stress. The study aimed to assess cadmium concentration in soil and leaves while also examining the soil composition in two grape varieties from Malayer city.

The findings showed that cadmium stress reduced chlorophyll content, relative water content, and increased ion leakage in grape plants. However, biochar treatment improved plant health by enhancing water retention capacity, chlorophyll, and phosphorus content while reducing ion leakage. In the soil, biochar altered the chemical forms of cadmium by reducing its exchangeable and carbonate fractions while increasing its binding to organic matter, oxides, and residual forms, making it less bioavailable.

Notably, biochar significantly reduced the availability of heavy metals compared to untreated soil. In the white Soltana grape variety, biochar application under 100 mg/kg cadmium stress led to a 48.4% increase in cadmium bound to organic matter compared to untreated samples, whereas the increase in the Perlet variety was 21.45%. Additionally, cadmium in the residual form increased by 34.97% in white Soltana and 30.34% in Perlet after biochar application. These results suggest that biochar effectively reduces cadmium bioavailability and toxicity, thereby mitigating the stress effects on grape plants.

An experiment led by Karaca [87] examined the effect of organic waste amendments—tobacco dust (TD), mushroom compost (MC), and grape marc (GM)—on the extractability of cadmium (Cd), copper (Cu), nickel (Ni), and zinc (Zn) in soil. The experiment involved soil incubation over six months and aimed to assess how these organic materials, which are rich in organic matter, influence the bioavailability of these metals within the soil matrix. Organic amendments were applied at concentrations of 0%, 2%, 4%, and 8% (in a moist state) relative to the mass of air-dried soil. The results indicated that the application of MC and GM significantly lowered soil pH, while all three organic amendments increased the organic matter content in the soil. Tobacco dust (TD) notably increased the levels of diethylenetriamine pentaacetic acid (DTPA)-extractable Cd and Cu, whereas GM and MC reduced Cd levels but increased Zn concentrations. Nickel levels were higher in soils amended with MC, likely due to the organic matter content in GM. The extractability of Cu decreased with the application of GM, but no consistent pattern was observed with MC. After six months, the concentrations of heavy metals were higher than those measured at the beginning of the experiment, possibly due to the degradation of organic matter and the subsequent release of metals.

Given the relative ease with which soil organic matter and pH can be modified, further investigation into their effects on metal extractability is warranted.

## 4. Grape Pomace-Derived Biochar for Pesticide Adsorption and Soil Enhancement

Fertilizers are typically derived from the aerobic decomposition of organic matter. However, research indicates that the extensive use of modern agricultural practices, including chemical fertilizers and pesticides, can deplete soil fertility and quality, thereby increasing environmental pollution risks. This can lead to the accumulation of toxic heavy metals in crops, potentially compromising food nutrition and edibility. Biological fertilizers, in contrast, offer an eco-friendly and cost-effective alternative with enhanced nutrient content. Grape pomace, rich in organic matter, has emerged as a valuable resource in agriculture, particularly for composting. When combined with soil amendments, grape pomace significantly influences the leaching dynamics of soil nutrients, with its high polyphenol content playing a key role in regulating nutrient release [88]. Additionally, grape pomace effectively aids in the removal of soil pesticides. As reported by Ohashi et al. [89], it can act as a hydrogen donor for anaerobic microorganisms, promoting the dechlorination of vinyl chloride monomer (VCM) and vinyl chloride (VC) to ethylene, thus detoxifying soil solutions. Marín-Benito et al. [90] also observed that grape pomace reduces the leaching of the non-mobile insecticide ‘Diazinon’ due to its cellulose and lignin adsorption properties. Additionally, composting with grape pomace not only enhances agricultural value but also contributes to the return of stable organic matter and nutrients to the soil, which may play a role in mitigating climate change by binding soil elements. However, the practical application of this solution must consider factors such as the organic content classification, energy supply, and water addition. Inadequate conditions may lead to odor emissions and poor-quality compost.

The behavior of pesticides in soils can be significantly influenced by the addition of biochar. However, studies on the effects of raw feedstock and biochar are limited.

The rate at which pesticides adhere to the surface of biochar is known as the kinetic adsorption phenomenon. In this process, pesticides transition from the bulk aqueous solution to the biochar surface and then permeate into the microporous and mesoporous structures of the material [91].

Yoon et al. [92] investigated the adsorption process and behavior of grape pomace biochar (GP-BC) for the removal of the pesticide cymoxanil (CM). The biochar was synthesized through pyrolysis at temperatures of 350, 550, and 750 °C, while batch tests were conducted to examine the impact of pyrolysis temperature, pH of the initial solution, and adsorption kinetics. GP-BC350 contained the highest amount of potassium (1.94%), the highest H/C ratio (0.905), the lowest surface area (0.25 m^2^/g), and the highest adsorption capacity for CM (161 mg CM/g BC) at pH 7. The results indicate optimal cymoxanil removal at low pyrolysis temperatures due to the retention of inherent adsorptive properties. Hydrophilic functional groups (such as carbonyl, hydroxyl, and carboxyl) and crystalline compounds (such as KCN and K_4_P_2_O_7_) were preserved in the biochar at lower temperatures, promoting adsorption through metal coordination and hydrophilic interactions. The results highlight GP-BC as a cost-effective, environmentally friendly adsorbent for pesticide elimination, providing a sustainable solution for eco-friendly waste management and resource conservation.

Gavrilas et al. [93] found that conditioning parameters for pomace at 30 °C resulted in the lowest pesticide content present in the final extract. Grape pomace can also be used to obtain extracts under different conditions.

Rodríguez-Cruz et al. [94] investigated the comparative adsorption of three pesticide groups—diazinon, linuron, and myclobutanil—by sewage sludge (SS), grape marc (GM), spent mushroom substrate (SMS), and residue-amended soils. The study assessed the impact of residue nature, soil type, pesticide properties, and the incubation period of residue–soil mixtures. Results showed that pesticide hydrophobicity did not dictate the adsorption process. Non-amended soils had distribution coefficients (Kd) ranging from 1.77–6.60 mL/g^−1^ for linuron, 0.54–5.52 mL/g^−1^ for diazinon, and 1.35–4.52 mL/g^−1^ for myclobutanil. In contrast, amended soils exhibited much higher Kd values—up to 4.8 times higher for linuron, 6.9 times higher for diazinon, and 5.3 times higher for myclobutanil. The highest Kd values were recorded for GM with diazinon and linuron and for SMS with myclobutanil. After 12 months of incubation, the Kd values for diazinon and linuron decreased, likely due to a reduction in organic carbon (OC), but no conversion of OC into more stable forms through humification was observed. Conversely, the Kd values for myclobutanil increased over the incubation period. The findings highlight the importance of considering multiple factors—soil type, pesticide properties, and residue characteristics—when applying pesticides and organic residues in agriculture to minimize environmental risks [95].

Table 3 summarizes the conditions and maximum absorption capacities for the absorption of pesticides.

According to Ohashi et al. [89], grape pomace can be used as a source of hydrogen for anaerobic micro-organisms for the purpose of dechlorination of tetrachloroethene (PCE), trichloroethene (TCE), dichloroethene (DCE), and vinyl chloride (VC) to ethylene for detoxification of vinyl chloride elimination in the soil solutions.

Marín-Benito [90] found grape pomace to be able to prevent leaching of diazinon through its lignin and cellulose. Notably, composting with grape pomace adds value to agriculture apart from organic matter and nutrient recycling in the solid form back into the earth, with the options of contributing to reducing climatic change through element sequestering in the earth.

The application of grape pomace extract prevents the toxic effects of diuron due to the antioxidant and hepatorenal protective activity of grape pomace [97].

Benkhemkhem et al. [96] studied the adsorption behavior of 2-mercaptobenzothiazole in aqueous solution on activated carbon derived from grape marc. The agricultural waste was treated chemically through acid treatment using phosphoric acid (40% H_3_PO_4_, 170 °C for 3 h), followed by pyrolysis at 650 °C for 2 h, to obtain GMAC-650 for use as an adsorbent. The obtained material was characterized using Fourier transform infrared spectroscopy (FTIR), X-ray diffraction (XRD), and Brunauer–Emmett–Teller (BET) analysis. Maximum adsorption (~99%) was achieved under optimum conditions: an adsorbent dose of 0.8 g/L, pH 8, an initial concentration of 10 mg/L, and a temperature of 45 °C. The adsorption kinetic data were analyzed using both the Langmuir and Freundlich isotherm equations. The results indicate that the non-linear approach is more accurate for estimating the isotherm parameters compared to the linear method.

Takeshita et al. [97] assessed the use of low-cost adsorbents—white grape bagasse, corn straw, peanut shell, and soybean hull—to replace activated carbon in removing the herbicides diuron and hexazinone from potable water. The water was spiked with herbicides at five different concentrations (1–5 mg/mL) and shaken with 0.1 g of each adsorbent. Adsorption was evaluated using the batch equilibrium method, and herbicide concentrations were measured by high-performance liquid chromatography (HPLC). The results indicated that diuron exhibited greater sorption than hexazinone, with Freundlich coefficients ranging from 2.99 to 11.93 mmol (1 − 1/n) L1/n kg^−1^ for diuron, compared to 2.31 to 4.61 mmol (1 − 1/n) L1/n kg^−1^ for hexazinone. The highest adsorption for diuron was observed in white grape bagasse (51.15%) and peanut shell (52.44%), while hexazinone was most strongly adsorbed in corn straw (22.77%) and white grape bagasse (21.48%). Overall, white grape bagasse showed great potential in adsorbing polar-type herbicides, which are known to pose significant risks to groundwater and surface water.

Similarly, Marín-Benito et al. [98] found that by adding organic matter to the soil, such as grape marc, it results in improved retention and reduced leaching.

Based on the research reviewed, grape pomace has shown great potential as an effective and eco-friendly solution for addressing environmental pollution, particularly in agricultural settings. The unique composition of grape pomace enables it to be used in various applications, such as composting, vermicomposting, pesticide removal, and as a biosorbent for toxic substances [99]. Studies have demonstrated that grape pomace not only improves soil quality by reducing pesticide leaching but also plays a significant role in removing harmful compounds from contaminated soil and aqueous solutions. Furthermore, grape pomace-derived biochar exhibits strong adsorption properties, particularly for pesticides, offering a sustainable approach to waste management and resource conservation.

## 5. Benefits and Drawbacks of Using Grape Pomace as a Biosorbent

**Figure 3:**
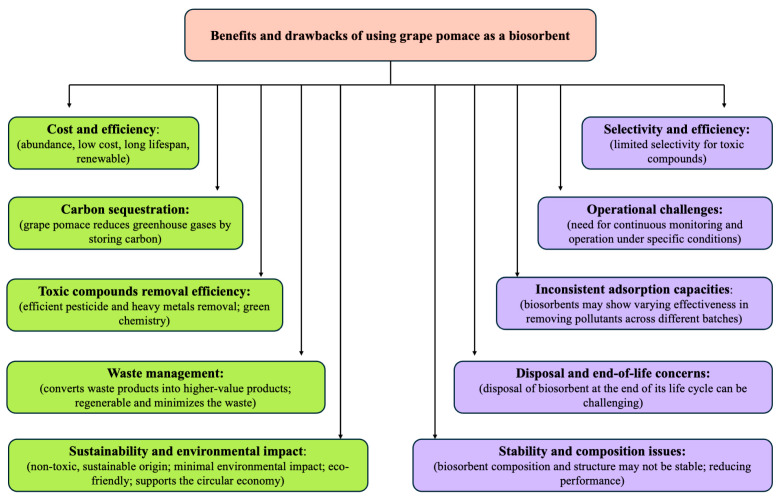
Advantages and disadvantages of using grape pomace as a biosorbent.

The studies reviewed highlight the effectiveness of grape pomace-based biosorbents in removing heavy metals and pesticides from liquid solutions and soil. Grape pomace offers several advantages, such as being a low-cost, sustainable option with high adsorption capacities. Its use can help prevent the release of toxic substances into the environment and mitigate its own environmental impact. However, the selection of an appropriate adsorbent depends on factors like the type of pesticide, operating conditions, and economic considerations, including production and operational costs. Each biosorbent, including grape pomace, has its strengths and weaknesses, and choosing the right one requires a clear understanding of the specific application needs (Figure 3).

The development of high-performance, cost-effective materials, such as grape pomace, is essential for meeting the agricultural sector’s demands. Grape pomace’s potential in biosorption could support the circular economy by addressing liquid solution pollution and reducing waste. While adsorption was selected as a preferred method due to its simplicity, low maintenance, cost-effectiveness, and lack of sludge production, the instability of biosorbents like grape pomace—compared to pure chemical substances—can cause inconsistent performance across batches.

## 6. Future Perspectives and Conclusions

The conversion of grape pomace, a byproduct of winemaking, into high-value-added products presents a promising alternative for waste utilization. Due to its unique composition, grape pomace has shown potential in generating a variety of valuable products across different sectors. Moreover, alternative biosorbents derived from grape pomace have proven to be highly effective in enhancing environmental, beverages, and soil safety by efficiently removing toxic substances.

Continued development and improvement of synthesis technologies for processing grape pomace are essential for scaling these processes and achieving more cost-effective solutions. This is key to advancing toward a circular economy, where grape pomace, as a waste resource, is used sustainably. Furthermore, the creation of adsorbents from grape pomace to effectively remove toxic and undesirable compounds will significantly enhance environmental safety, contributing to the broader goal of sustainable resource utilization.

The need for further research is critical to understanding the influence of different types of grape pomace and how they are influenced by grape varieties and winemaking processes. Continuous assessment of environmental impact and cost-effectiveness will be essential for the sustainable use of grape pomace in removing contaminants. Despite its potential, more studies are required to explore its practical applications, ensuring compliance with environmental regulations and enhancing its reliability for industrial use.

## Figures and Tables

**Table 2:** Comparison of carbonaceous materials produced from waste grape pomace as adsorbent for heavy metals from aqueous solution.

Biomass	Thermal Process	Carbonaceous Materials	Contaminants	Conditions	Initial Concentration	Maximum Adsorption Capacities	Observations	Adsorption Isotherms	References
Merlot grape marc	Biorefinery	Raw feedstock	Pb^2+^	pH 5.5, 22 °C	20 mg/L	40.14 mg/g	-	Langmuir, non-linear	[53]
Sauvignon Blanc grape marc	Biorefinery	Raw feedstock	Pb^2+^	pH 5.5, 22 °C	20 mg/L	63.76 mg/g	-	Langmuir, non-linear	[53]
Grape pomace	Hydrothermal	KOH-activated hydrochar	Pb^2+^	pH 5	40–180 mg/L	137 mg/g	-	Langmuir, Freudlich and Sips	[54]
Grape pomace	Pyrolysis	Biochar	Pb^2+^	700 °C, 0.5 h	300 mg/L	300 mg/L	-	Langmuir, Freudlich and Temkin	[55]
Grape pomace	Pyrolysis	Biochar	Cd^2+^	Magnetization	43 mg/L	-	Even when multiple metals were present,	-	[58]
Grape pomace	Oven-dried	Raw feedstock	Pb^2+^, Cd^2+^	pH 7.0 for Cd^2+^; pH 3.0 for Pb^2+^	5–600 mg/L	-	Pb^2+^ showed a slower but higher adsorption affinity than Cd^2+^	Langmuir, linear	[64]
Grape pomace	Oven-dried	Raw feedstock	Pb^2+^, Cd^2+^	pH 5, 45 °C, 2 h	300 mg/L	357.14 mg/g—Pb^2+^, 156.25 mg/g—Cd^2+^	-	Langmuir and Freundlich	[20]
Grape pomace	Oven-dried	Raw feedstock	Pb^2+^, Cd^2+^	pH 5.5, 20 °C	0.05 mmol/L—Pb^2+^ 0.07 mmol/L—Cd^2+^	0.24 mmol/g for Pb^2+^, Cd^2+^	The presence of NaCl and NaClO_4_ reduced metal sorption	Langmuir and Freundlich	[66]
Grape pomace	Pyrolysis	Biochar	Pb^2+^, Cd^2+^	300 °C, 450 °C, 600 °C, pH > 3	5–1000 mg/L	250.81, 327.31, 377.36 mg/g for Cd^2+^, 156.12, 237.25, 258.86 mg/g for Pb^2+^.	-	Langmuir and Freundlich	[68]
Grape pomace	Carbonization	Raw feedstock	Pb^2+^, Cd^2+^	pH 5.5—Pb^2+^, pH 6—Cd^2+^, 800 °C, 1.75 h	1.31 mg/L	98%	-	Langmuir and Freundlich	[69]
Wine processing waste sludge	Air-dried	Raw feedstock	Ni	50 °C	30, 45, 60, 75, 90 mg/L	66.55 μmol/g	-	Langmuir and Freundlich	[70]
Grape bagasse	Dried	H_3_PO_4_ -activated	Cu^2+^	-	10–100 mg/L	43.47 mg/g		Langmuir, Freundlich, Temkin and Dubinin–Radushkevich	[85]
Grape bagasse	Pyrolysis	Char	Cu^2+^	pH 5.5, adsorbent dose of 1.5 g/L	30, 50, 80, 120, 200 mg/L	42 mg/g	Char synthesized at 700 °C is most effective	Langmuir and Freundlich	[72]
Grape waste	-	Gel	Cr^6+^	pH 4	-	1.91 mol/kg	The adsorption capacity increases with increasing solute concentration	Langmuir	[73]
Grape bagasse	Pyrolysis	Char	Hg^2+^	pH 4, 40 °C	100 and 200 mg/L	45.9 mg/g	-	Langmuir	[75]
Green grape marc	Sun-dried	Raw feedstock	Hg^2+^	pH 5–7	78 mg/L	36.39 mg/g	-	Langmuir, Freundlich, Temkin, and D-R	[76]
Grape pomace	Pyrolysis	Biochar	Ni^2+^, Zn^2+^	1 h		0.2 mg/L of Ni^2+^, 2.0 mg/L of Zn^2+^	-	-	[77]
Isabel grape bagasse	Freeze-dried	Raw feedstock	Zn^2+^	pH 3–5, 22 °C, 20 mg biosorbent mass	100 mg/L	192.3 mg/L at pH 3 246.3 mg/L at pH 5	-	Langmuir and Freundlich, linear	[78]
Cabernet Sauvignon grape pomace	Dried	Raw feedstock	Cd^2+^, Ni^2+^, Co^2+^, Pb^2+^	24 h, 10 g/L biosorbent	1 mmol/L	0.34 mg/g Cd^2+^, 0.54 mg/g Ni^2+^, 0.28 mg/g Co^2+^, 15.12 mg/g Pb^2+^	-	-	[80]

**Table 3:** Comparison of carbonaceous materials produced from waste grape pomace as adsorbent for pesticides.

Biomass	Thermal Process	Carbonaceous Materials	Contaminants	Conditions	Maximum Adsorption Capacities	Observations	References
Grape pomace	Pyrolysis	Biochar	Cymoxanil	350 °C, 2 h 550 °C, 2 h 750 °C, 2 h	161.02 mg CM/g BC for 350 °C 77.57 mg CM/g BC for 550 °C 45.64 mg CM/g BC for 750 °C	-	[92]
White grape pomace	Refrigeration Dried	-	-	30 °C	the lowest pesticide content present in the final extract	grape pomace can be used as well for obtaining extracts with different conditions	[93]
Grape marc	Air-dried	Raw feedstock	Diazinon Linuron Myclobutanil	1 and 12 months outdoor incubation	highest adsorption of linuron and diazinon	showed the highest adsorption of diazinon, linuron	[94]
Muscat Bailey wine pomace extract	-	Extract	tetrachloroethene (PCE), trichloroethene (TCE), dichloroethene (DCE), vinyl chloride (VC)	Microbial degradation with Dehalococcoides spp, pH 7–8	-	lactic acid, and tartaric acid in wine pomace extract function as hydrogen donors in the anaerobic microbial degradation of chloroethene	[89]
Grape pomace	-	Raw feedstock	Diazinon Linuron Myclobutanil	-	-	grape pomace prevents diazinon from leaching through its lignin and cellulose	[90]
Grape marc	Pyrolysis	H_3_PO_4_—activated biochar	2-mercaptobenzothiazole	pH = 8, 45 °C, adsorbent dose 0.8 g/L	99%	-	[95]
White grape bagasse	-	Raw feedstock	Diuron hexazinone	0.1 g quantity of grape bagasse was added to 10 mL of potable water contaminated with herbicide	51.15% 21.48%	great potentials in the adsorption of polar-type herbicides	[96]

## Data Availability

No new data were created or analyzed in this review. Data sharing is not applicable to this paper.
